# Supplementary figures and images for: Association between the proportion of laparoscopic approaches for digestive surgeries and the incidence of consequent surgical site infections, 2009–2019: A retrospective observational study based on national surveillance data in Japan
Source: PLoS One. 2023 Feb 17;18(2):e0281838. doi: 10.1371/journal.pone.0281838 (PMC9937488; doi:10.1371/journal.pone.0281838)

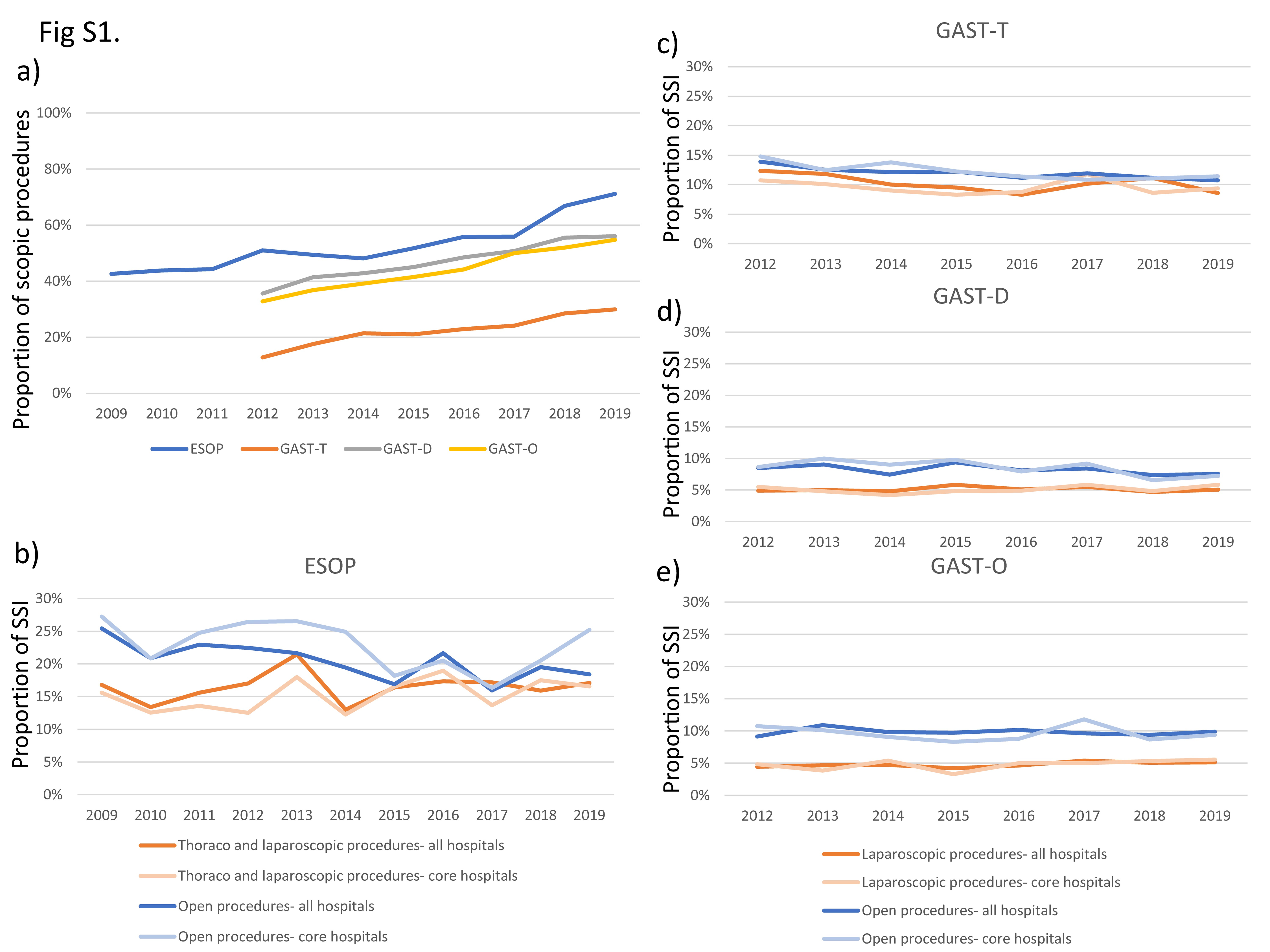

Supplement: S1 Fig — a) The proportion of thoracoscopic and laparoscopic procedures for esophageal (ESOP) and three types of gastric surgery (total gastrectomy: GAST-T, distal gastrectomy: GAST-D, other gastrectomy: GAST-O). The blue line shows the rate of ESOP in the core participating hospitals. The orange line shows the rate of GAST-T. The gray line shows GAST-D. The yellow line shows the rate of GAST-O. b-e) The blue line shows the surgical site infection rate for open procedures in all participating hospitals. The light blue line shows the surgical site infection rate for open procedures in the core participating hospitals. The orange line shows the surgical site infection rate for laparoscopic procedures in all participating hospitals. The light orange line shows the surgical site infection rate for laparoscopic procedures in the core participating hospitals. (TIF) [file pone.0281838.s001.tif]

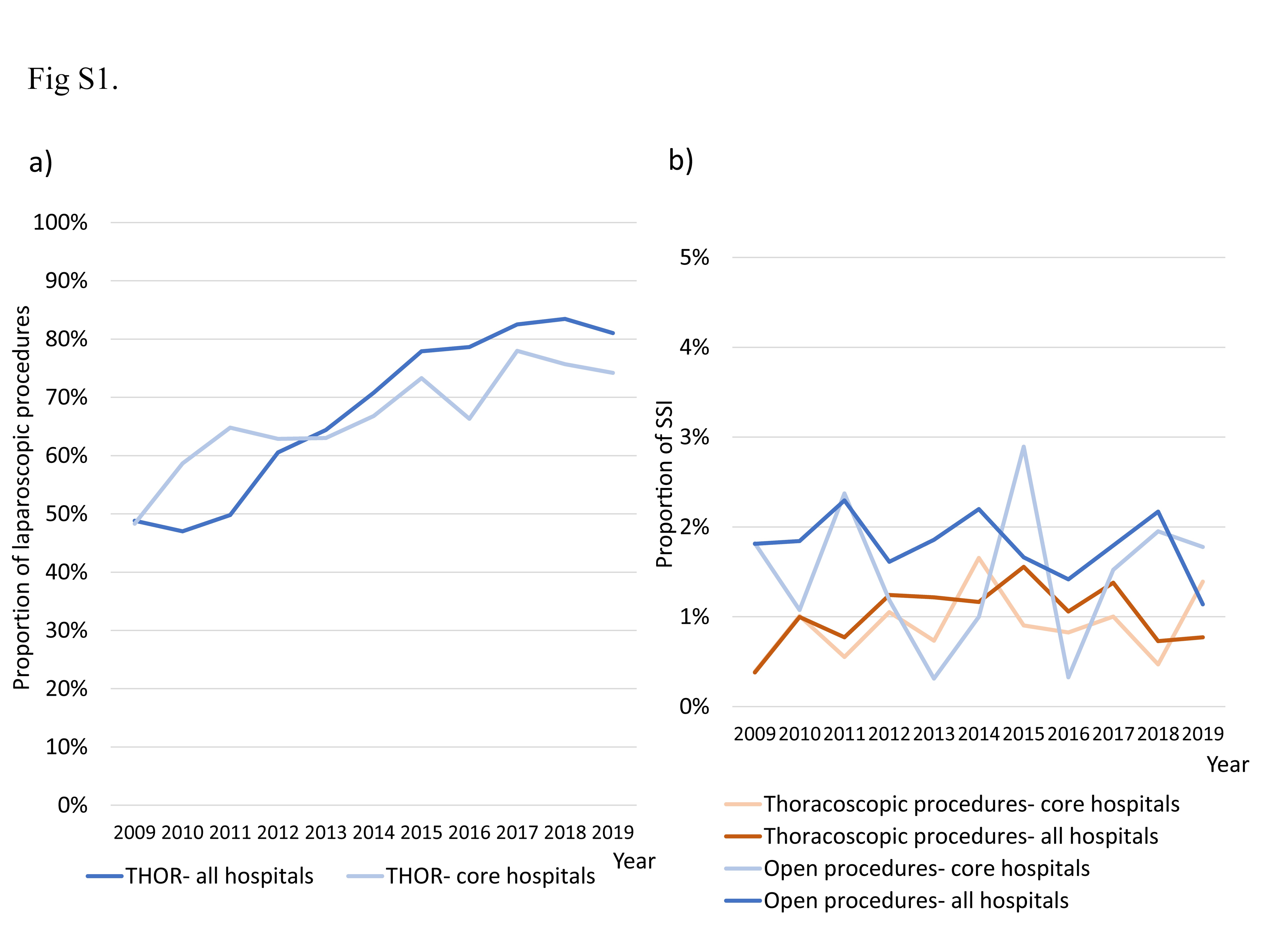

Supplement: S2 Fig — a) The proportion of thoracoscopic procedures for thoracic surgery (THOR). The blue line shows the rate of thoracoscopic surgery in all participating hospitals and the light blue line shows the rate in the core participating hospitals. b) The blue line shows the surgical site infection rate for open procedures in all participating hospitals. The light blue line shows the surgical site infection rate for open procedures in the core participating hospitals. The orange line shows the surgical site infection rate for thoracoscopic procedures in all participating hospitals. The light orange line shows the surgical site infection rate for thoracoscopic procedures in the core participating hospitals. (TIF) [file pone.0281838.s002.tif]
